# 定量影像学在肺癌放/化疗疗效评估中的应用

**DOI:** 10.3779/j.issn.1009-3419.2017.06.07

**Published:** 2017-06-20

**Authors:** 玉新 焦

**Affiliations:** 1 200040 上海，复旦大学附属华东医院肿瘤放疗科 Department of Radiology Oncology, Shanghai 200040, China; 2 200040 上海，华东医院影像科 Department of Radiology, Shanghai 200040, China; 3 200040 上海，华东医院张国桢肺小结节诊疗中心 Zhang Guozhen Diagnosis and Treatment Center of Micronodular Lung Cancer (DTC-MLC), Fudan University Huadong Hospital, Shanghai 200040, China

**Keywords:** 肺肿瘤, 疗效, 定量分析, 影像学, Lung neoplasms, Response evaluation, Quantitative Imaging, Chemoradiation

## Abstract

精准医疗的实施要求及时准确地对治疗疗效进行评估，以便于治疗方案的调整和优化，从而进一步提高疗效，改善预后。以定量评估为基础的影像组学以其无创、直观和可重复的特点在临床疗效评估方面具有不可替代的作用。本文将综述定量影像学在肺癌放化疗疗效评估中的应用现状及其相关进展。

## Abstract (en)

Precision medicine demands accurate assessment of tumor response to therapies with the purpose of timely optimization or adjustment of the given treatment regimens. Chemoradiation remains the standard of care in advanced lung cancers and imaging-based noninvasive response evaluation could improve therapeutic efficacy and reduce treatment-related severe side effects. In this review, we overviewed the applications and pitfalls of major imaging modalities in response evaluation in lung cancer from a quantitative perspective. The development of radiomics will provide more precise information for treatment predicting, evaluation and prognosis.

根据2015年中国癌症最新统计数据，肺癌的发病率和致死率均居首位^[1]^。目前，外科手术、放射治疗、化学治疗和靶向治疗是肺癌的主要治疗手段，免疫治疗、生物治疗等也为患者提供了更多治疗选择。在精准医疗时代，治疗方案的精准选择非常重要，而及时、准确的疗效评估对于及时调整治疗策略以及改善预后等的作用同样重要。随着计算机断层扫描（computed tomography, CT）、磁共振成像（magnetic resonance imaging, MRI）、B超、正电子发射型计算机断层显像（positron emission computed tomography, PET）/CT等成像技术的不断发展以及图像后处理技术的不断优化，以定量影像为特征的影像组学为肺癌的疗效评估提供了更为完善和可靠的方法。本文将简要综述目前临床上肺癌放化疗后疗效评估的影像学定量参数及其相关进展。

## 1 主要影像学疗效评估标准发展简述

目前临床上肺癌疗效评估主要依据基于解剖形态学变化和功能代谢变化的实体瘤疗效评估标准。前者主要包括世界卫生组织（World Health Organization, WHO）标准，实体瘤疗效评价标准（Response Evaluation Criteria in Solid Tumors, RECISTs）1.0与RECIST 1.1。通过双径测量法（WHO标准）或者单径测量法（RECIST 1.0/RECIST 1.1）将疗效反应分为4级：完全缓解（complete response, CR）、部分缓解（partial response, PR）、疾病稳定（stable disease, SD）和疾病进展（progressive disease, PD）。RECIST 1.1标准现已成为国内外实体肿瘤（包括肺癌）疗效评估的主要参考标准^[2, 3]^。功能代谢相关标准依赖于核医学分子影像为代表的成像技术，从代谢和功能方面反映肿瘤的变化，能在肿瘤出现可测量的体积变化前检测肿瘤对治疗的反应，从而可以指导医生更早地调整或优化治疗方案。主要包括欧洲肿瘤研究和治疗组织（European Organization for Research and Treatment for Cancer, EORTC）标准和实体瘤治疗疗效PET评估标准（PET Response Criteria in Solid Tumors, PERCIST）。通过比较病灶标准摄取值（standardized uptake value, SUV）和瘦体重标准摄取值（SUV normalized to lean body mass, SUL）的变化将肿瘤分为完全代谢反应（complete metabolic response, CMR）、部分代谢反应（partial metabolic response, PMR）、代谢稳定（stable metabolic disease, SMD）和代谢进展（progressive metabolic disease, PMD）4种状态。与EORTC标准比较，PERCIST标准在靶病灶的选择上有明确的原则，测量评价指标更丰富，包括SUL及糖酵解总量（total lesion glycolysis, TLG）等，因而临床操作性更强^[4]^。

## 2 不同定量影像学参数在肺癌疗效评估中的应用及进展

### 2.1 CT

由于肺部存在天然的空气对比，CT成为评估肺部病变最主要的影像学检查手段。此外，CT成像速度快，可以最大幅度地降低呼吸和心脏运动所致伪影对图像质量的影响，因此被广泛应用于肺癌疗效评估。除WHO和RECIST 1.0/1.1标准参数外，还有其它诸多定量参数（表 1）。

**1:** 常用CT定量参数及其特点 Commonly used quantitative metrics in CT imaging

Category	Parameters	Software	Characteristics
Volume	3D Volume	MEDx TDA	1. Used to measure overall tumor changes. 2. Good reproducibility, especially in those tumors with irregular shapes. 3. Lack of well-accepted standards among various processing algorithms.
Density	CT value SD RER	AW PACS ITK-SNAP	1. Capable of assessing intratumoral changes. 2. Time-consuming in comparison to traditional anatomical and morphological measurements. 3. Intraobserver and interobserver variations as well as variations due to measurement approaches.
Texture	ASM Entropy Contrast	OneCut graph ImageJ^®^ MaZda^®^	1. Advantage of quantitative description of tumor heterogeneity. 2. Measurement outcome less subject to observers and methodologies. 3. Results vulnerable to scanning equipment and post-processing algorithms.
Perfusion	BF, BV, TEF MTT, PS PBV, TVV	Syngo 2008G AW Functional CT	1. Able to provide functional imaging information by assessment of vascular perfusion within tumors, which may be more accurate for evaluation of physiological changes than dimensional or volumetric methodologies, such as RECIST. 2. Be aware of the radiation dose. 3. Performance could be improved by optimization of hemodynamics models.
Energy	IRA, IU, AEF	Liver VNC, Syngo IPIPE	1. Material decomposition, especially iodine quantitation, provides biological and chemical information related to tumors, in a manner similar to PET-CT imaging. 2. Thanks to the virtual non-contrast technique, the unenhanced examination could be waivered, hence radiation dose reduced. 3. Image quality may be compromised in patients with high BMI values. 4. Unclear or inconsistent relationship between disease conditions and measurements.
RER: relative enhancement ratios; SD: standard deviation of CT value; ASM: angular second moment; BF: blood flow; BV: blood volume; TEF: total tumor extravascular flow; MTT: mean transit time; PS: capillary permeability surface area product; PBV: Pallak blood volume; TVV: total tumor vascular volume; IRA: iodine-related attenuation; IU: iodine uptake; AEF: arterial enhancement fraction; TDA: three-dimensional algorithm; AW: Advanced workstation; PACS: picture archiving and communication system; VNC: virtual non-contrast.			

#### 2.1.1 三维体积定量参数

传统的RECIST标准局限于二维平面测量肿瘤最长径，不能反映肿瘤的整体。近年来CT三维多平面重组成像技术（multiplanar reconstruction, MPR）的发展弥补了常规横断面显像无法对病灶进行全面整体观察的缺陷，利用MPR技术可以从冠状面、矢状面及任意角度、层面观察肺癌病灶，从整体的角度获取病灶所有的形态学数据，从而能够更全面、更精确地评估肿瘤的变化。多项研究证实三维体积测量优于单纯的径线测量，能够更加精准、更加早期地反映肺肿瘤的变化^[5-7]^，对及时调整治疗方案具有重要意义。而且体积测量法具有更好的重复性，尤其是对于那些边界不规则或者呈融合状的肺肿瘤，体积测量法更加适用^[8]^。但由于容积测量相对耗时、费力，并未常规应用于临床工作中。

#### 2.1.2 密度定量参数

肺癌对药物的反应或可表现为肿瘤内部成分的改变（出血、坏死、血管抑制或空洞形成等），而且这种改变早于病灶整体形态的变化^[9]^，此时若仍以形态学变化为评判标准，可能会错误地估计治疗效果，影响治疗决策。病灶的CT值反映了物质对X射线的衰减程度，可在一定程度上反映肿瘤内部成分的变化。在M.D. Anderson癌症中心进行的一项关于转移性胃肠道间质瘤疗效评估的研究中，作者将肿瘤密度与病灶最大径变化相结合，共同作为疗效评估的参考指标，提出了新的实体瘤评价标准（new response criteria, NRC）：病灶最大径减小10%或者病灶的平均CT值减小15%作为部分缓解标准，具有较高的敏感性与特异性^[10]^。Lee等^[11]^通过研究80例非小细胞肺癌（non-small cell lung cancer, NSCLC）患者经表皮生长因子受体酪氨酸激酶抑制剂（epidermal growth factor receptor tyrosine kinase inhibitors, EGFR-TKI）治疗前后的影像学资料，以肿瘤内部实性成分变化为基础，参照上述NRC标准，发现20%经RECIST标准评估为治疗无反应的肿瘤根据NRC新标准则为局部缓解，并且新标准与患者总体生存率存在明显的统计学相关性。

另外，病灶密度变化对肿瘤复发与放疗所致放射性损伤的鉴别也有一定的价值。Mattonen等通过随访22例经立体定向放射治疗的早期NSCLC患者的影像表现，发现病灶实变区域平均CT值以及磨玻璃样变区域的CT值标准差具有鉴别放射性损伤与复发的潜能，复发者实变区域的CT值以及磨玻璃样变区域的CT值标准差均高于放射性肺损伤者，与RECIST和三维容积测量标准相比，其出现差异的时间更早（9个月*vs* 15个月，*P*=0.007, 8）^[12]^。

#### 2.1.3 图像纹理定量参数

图像纹理特征分析利用特定的算法对数字图像内部的像素分布特征进行提取和参数量化分析。临床中常见的医学影像学图像均可进行纹理特征分析，其在一定程度上反映肿瘤内部的异质性，可用于多种肿瘤的良恶性鉴别、定性诊断及病理分级等方面^[13, 14]^。多项研究^[15-17]^表明，基于纹理分析的定量参数在肺癌疗效评估中具有潜在应用价值，有可能成为新的疗效评估预测影像学指标。尤其是在肺癌治疗后的随访过程中，定量纹理参数与形态学指标相比，能更早更准确地筛选出早期复发的高危患者，其准确度高达81.8%，从而有助于及时调整治疗策略，改善患者预后^[18]^。

#### 2.1.4 灌注CT定量参数

CT灌注成像借助数学模型与专用软件，计算造影剂通过血管进入组织的血流动力学相关定量参数（如血容量、血流量、表面通透性、峰值时间等），在获得形态学数据的同时评估肿瘤的微循环状态^[19]^。多项研究^[20-22]^结果表明，灌注CT影像在定量评估肺癌的治疗疗效方面具有潜在价值。一项关于肺癌化疗疗效评价的*meta*分析发现80%的研究均发现治疗有效者的血流量明显下降^[22]^。总体来讲，肺癌灌注CT参数能反映肿瘤内部微循环的变化，在肺癌疗效评估中具有一定前景，特别是对于接受抗血管生成药物治疗的患者；但不同的扫描条件，不同的灌注分析模型之间尚无统一标准，数学模型的构建对分析结果的影响较大^[23]^，其重复性有待提高。此外，较高的辐射剂量和造影剂用量及其相关毒性亦是影响灌注扫描应用的因素。

#### 2.1.5 能量（或能谱）CT定量参数

能量CT通过一次扫描可以重建获得多组基于单能量X线的CT图像，根据不同能量的射线穿过同一物质时衰减系数不同，可以计算物质内的成分构成，其碘图更可直观地反映肿瘤内部的碘分布和含量^[24]^，间接反映肿瘤的内部血供和微环境状态，因此在评估肿瘤治疗疗效方面具有一定价值。Baxa针对肺癌患者的双能量CT研究显示，在缓解组，肺癌原发病灶及转移淋巴结的碘摄取值均明显降低，而在未缓解组则无明显下降甚至呈持续上升趋势^[25, 26]^。另有研究表明肺癌碘定量参数与肿瘤血管内皮生长因子（vascular endothelial growth factor, VEGF）密切相关^[27]^，而且无论是肺癌病灶还是PET/CT阳性淋巴结，其最大碘相关衰减值均与最大标准化摄取值（maximum standardized uptake value, SUV_max_）存在显著相关性^[28]^。能量CT除了具有一定程度的功能影像特征外，其成像速度快，检查费用低，更能满足临床治疗疗效评估对影像检查快捷和高频次的要求，在评估肿瘤治疗疗效方面具有十分重要的临床应用价值。

### 2.2 MRI

MRI具有无辐射、任意平面成像、多序列采集等优点，随着MR成像技术的迅速发展，其在肺癌诊断、分期、疗效评估等方面中的应用越来越受到重视，尤其是弥散加权成像和动态增强成像将肺癌疗效评估带入了分子功能水平。

#### 2.2.1 动态增强MR成像（dynamic contrast-enhanced MRI, DCE-MRI）定量参数

静脉注射顺磁性对比剂后，采用T1加权成像，对造影剂的浓度变化进行追踪分析，形成时间信号强度曲线，运用药代动力学模型，获得反映肿瘤内部微循环状态的定量参数，主要包括：早期强化峰值、最大强化率、最大强化斜率、容量转移常数（volume transfer constant, K^trans^）、速率常数（rate constant, K_ep_）血管外细胞外间隙容积比（Ve）等，多项研究证明上述参数可作为评估肿瘤（包括肺癌）放化疗疗效的定量指标^[29-31]^，一项随机双盲临床试验发现，DCE-MR定量参数用于评估肺癌骨转移患者的疗效也具有一定潜能^[32]^。de Langen等^[33]^比较了CT、PET/CT和DCE-MRI监测NSCLC疗效的效能，发现后两者的时效性远优于CT。

#### 2.2.2 弥散加权MR成像（diffusion-weighted MRI, DWI-MRI）定量参数

DWI-MRI是利用水分子布朗运动的原理，以表观弥散系数（apparent diffusion coefficient, ADC）定量分析水分子弥散受限的程度，通过灰度对比直观显示感兴趣区内的水分子弥散状况。恶性肿瘤因其内部水分子活动受限，ADC值较低，在DWI图像上常显示为明显的高信号。相比于DCE-MRI，DWI-MRI在肺癌中的应用更为广泛，涉及到肺部肿瘤的良恶性鉴别、肺癌临床分期、术后肺功能评估、治疗效果评价及预后预测等各方面^[34, 35]^。在Yabuuchi等^[36]^的研究中，28例NSCLC患者于化疗前与化疗后3周-4周行DWI-MRI，化疗前与化疗后6-8行胸部CT检查结果显示，将ADC值变化同肿块大小比较，结果发现治疗早期ADC的变化程度与最终肿瘤的退缩程度明显相关，并且ADC值的变化要早于肿瘤形态的发生，Xu等^[37]^分析了192例NSCLC患者同步放化疗中与放化疗后DWI-MRI图像中ADC值与基线值的变化，同样发现治疗中与治疗后ADC值变化率与肿瘤退缩率呈明显正相关性，甚至有研究认为DWI-MRI肺癌疗效评估能力优于PET/CT^[38]^。这些研究明确表明ADC定量参数可作为肺癌临床治疗疗效早期评估与预测预后的功能学参考指标，有助于临床医生及时发现疗效不佳的患者，进而改变治疗策略。但是ADC值易受*b*值、场强、呼吸运动、脉搏等多种因素的影响，需要解决ADC值随访的稳定性、标准化等问题。体素内不相干运动扩散加权成像（intravoxel incoherent motion diffusion weighted imaging, IVIM-DWI）应运而生，其以ADC值为基础，将组织水分子扩散与血流灌注分离，并用不同的参数表示，能够更加真实地反应肿瘤对治疗的反应状态。有研究认为，其参数（f, D）在鉴别肺部良恶性病变方面具有重要意义^[39, 40]^。对于接受体部立体定向放射治疗（stereotactic body radiation therapy, SBRT）的肺癌患者，局部容易呈现肿块状或弥漫性改变，此时，肿瘤复发进展与良性反应性改变的鉴别尤为重要，上述研究结果为IVIM-DWI应用于肺癌疗效监测评估（尤其是SBRT治疗的患者）奠定了基础。有研究称全身扩散加权成像联合使用短反转恢复序列（short-T1 inversion recovery, STIR）用于淋巴结或远处转移的评估有一定优势，其准确性高（66%-88%）^[35]^，有较广阔的临床应用前景。

### 2.3 B超

放化疗联合治疗为局部晚期NSCLC患者的标准治疗方案，有研究证实手术切除降期患者的残余病灶能提高患者的总生存^[41]^，因此放化疗后的疗效评价（重新分期）对此类患者的筛选尤为重要，经气管或食管腔内超声引导针吸活检技术（endoscopic bronchial/ esophageal ultrasound-guided fine needle aspiration, EBUS/EUS-FNA）以及气道内超声弹性成像技术，具有微创、并发症少、无需全身麻醉，适应证较广泛等诸多优点，多项研究^[42, 43]^证实其用于肺癌疗效评价（纵隔重新分期）具有较高的准确性（83.1%-86.8%），更有研究^[44]^认为内镜超声可替代PET/CT用于局部晚期肺癌患者放化疗后的重新分期，为临床医生筛选合适的患者提供了重要参考依据。但是内镜超声属于一种侵入性检查，患者接受度尚待提高，难以实现重复多次检查以满足疗效评价的时效性需求。

### 2.4 PET/CT

PET/CT成像利用特异性显像剂，从分子生物学水平定性及定量揭示肿瘤的发展及转归，已经广泛应用于良恶性肿瘤鉴别、临床分期、疗效评估、随访监测等各个方面，一定程度上是目前评估肿瘤治疗疗效的影像学金标准^[45]^。

#### 2.4.1 标准摄取值

关于PET/CT在疗效评估中的应用，众多的研究集中于肿瘤SUV的变化^[46, 47]^，治疗前后SUV变化率可作为疗效评估的定量指标。EORTC标准将SUV_max_降低百分数≥25%（PERCIST标准中SUL_peak_降低百分数≥30%）作为评价实体瘤代谢缓解与否的标准^[4]^。需要指出的是，SUV_mean_/SUV_max_/SUL_peak_均基于感兴趣区内的单一体素分布，但是恶性肿瘤的异质性明显，故而此类定量参数不一定能准确地反映肿瘤治疗后的整体变化情况；此外，SUV易受给药剂量、给药-扫描间隔时间、扫描重建参数、患者血糖水平、器官运动等生物学以及物理学多重因素的影响，阈值差异性较大，进而影响治疗反应评价的准确性，用于疗效评估的稳定性需进一步研究探索^[48-50]^。

#### 2.4.2 代谢体积定量参数

鉴于SUV的测量存在诸多不足和缺乏对肿瘤整体情况的反映，基于体积的定量代谢参数成为新的研究目标，主要参数包括代谢肿瘤体积（metabolic tumor volume, MTV）和总病灶糖酵解（total lesion glycolysis, TLG）。多项研究已经证实代谢体积参数在肺癌的疗效评估中的表现优于单纯SUV变化^[51-53]^。RTOG0235临床研究分析了PET/CT定量参数在局部晚期肺癌患者预后评估中的价值，发现MTV是总生存期（overall survival, OS）的独立影响因素，高MTV患者预后较差，局部失败率较高，而SUV_max_与预后无关^[51]^。另一项针对28例接受同步放化疗的NSCLC进行的研究^[52]^中，患者分别在治疗前、治疗中第2个周末、治疗结束后第2个周末，治疗结束后第3个月时接受PET/CT检查，结果显示治疗前TLG低于500，治疗前后TLG下降超过38%的患者具有较长的无进展生存期（progression-free survival, PFS）。Satoh等^[53]^对88例I期NSCLC患者在SBRT前行PET/CT检查，对比分析定量参数SUV_max_、MTV、TLG50、TLG60在预后评估中的价值，发现上述定量参数均与无病生存期（disease-free survival, DFS）相关；但当肿瘤大于3 cm时，SUV_max_与预后无关，其主要原因可能是肿瘤越大异质性越明显，基于采样体素的SUV_max_反映肿瘤整体状态的性能越差。

#### 2.4.3 非FDG显像在肺癌疗效评估中的应用

新型影像示踪剂的应用可特异性反映恶性肿瘤的氧合状态、活性细胞增殖程度，这对治疗效果评估及方案及时优化具有重要临床意义。其中胸腺嘧啶（^18^F-fluorothymidine, ^18^F-FLT）是最常用的核苷酸类代谢显像剂，能反映细胞增殖活性。有研究^[54]^显示，与^18^F-FDG相比^18^F-FLT能更早期评价治疗反应。多种示踪剂可用于乏氧显像，较早的是氟硝基咪唑（^18^F-fluoromisonidazole, ^18^F-FMISO），其可选择性结合乏氧细胞，反映肿瘤乏氧状况。Eschmann研究发现肿瘤肌肉比值和肿瘤纵隔比值较高的患者容易出现复发，预后较差，若及时评估氧合状态，发现乏氧区域，采取使用增敏剂或其它相应临床措施，有可能改善患者预后提高生存率。由于^18^F-FMISO累积速度慢，肿瘤/背景比值较低，需要延迟扫描，相继出现了硝基咪唑阿拉伯糖苷（^18^F-fluoroazomycin arabinozide, ^18^F-FAZA）、甲基氨基硫脲（^64^Cu-methylthiosemicarbazone, ^64^Cu-ATSM）等多种乏氧显像剂，其中^64^Cu-ATSM因具有较理想的生物学分布、良好的肿瘤背景比、适宜的半衰期等优点，被广泛应用于各种肿瘤的乏氧显像中（包括肺癌）^[55]^，但是显像与肿瘤实际乏氧状态的对应关系，扫描时机仍不明确，需要进一步探索。

总之，及时准确的肺癌疗效评估有助于早期发现患者对治疗的反应状态，进而调整优化治疗方案，实现更为精准和个体化的治疗。CT、MRI、EBUS/EUS、PET/CT定量参数对于肺癌疗效评估各方面有重要的指导意义，与单一形态学参数相比，功能影像定量参数在反映肿瘤的生物学特点方面更有优势，更能满足现代肺癌治疗学的发展需求。但是目前各种定量参数的应用仍在探索和规范之中，形成标准化的应用方案仍需时间，下一步工作需优化现有的算法与物理模型，将多种影像学定量参数相互融合，综合判断，使无创性定量影像在肺癌精准治疗临床实践中发挥更大作用。实现影像定量数据与生物学、基因学数据的关联是将来以影像为中心的无创疗效评估的关键，我们相信大数据分析和影像组学的发展将为此提供最终解决方案。

## References

[1] Chen W, Zheng R, Baade PD (2016). Cancer statistics in China, 2015. CA Cancer J Clin.

[2] Therasse P, Arbuck SG, Eisenhauer EA (2000). New guidelines to evaluate the response to treatment in solid tumors. European Organization for Research and Treatment of Cancer, National Cancer Institute of the United States, National Cancer Institute of Canada. J Natl Cancer Inst.

[3] Eisenhauer EA, Therasse P, Bogaerts J (2009). New response evaluation criteria in solid tumours: revised RECIST guideline (version 1.1). Eur J Cancer.

[4] Wahl RL, Jacene H, Kasamon Y (2009). From RECIST to PERCIST: Evolving Considerations for PET response criteria in solid tumors. J Nucl Med.

[5] Force J, Rajan A, Dombi E (2011). Assessment of objective responses using volumetric evaluation in advanced thymic malignancies and metastatic non-small cell lung cancer. J Thorac Oncol.

[6] Hayes SA, Pietanza MC, O'Driscoll D (2016). Comparison of CT volumetric measurement with RECIST response in patients with lung cancer. Eur J Radiol.

[7] Lee J, Lee J, Choi J (2015). Early treatment volume reduction rate as a prognostic factor in patients treated with chemoradiotherapy for limited stage small cell lung cancer. Radiat Oncol J.

[8] Mozley PD, Bendtsen C, Zhao BS (2012). Measurement of tumor volumes improves RECIST-Based response assessments in advanced lung cancer. Transl Oncol.

[9] Coche E (2016). Evaluation of lung tumor response to therapy: current and emerging techniques. Diagn Interv Imaging.

[10] Choi H, Charnsangavej C, Faria SC (2007). Correlation of computed tomography and positron emission tomography in patients with metastatic gastrointestinal stromal tumor treated at a single institution with imatinib mesylate: proposal of new computed tomography response criteria. J Clin Oncol.

[11] Lee HY, Lee KS, Ahn MJ (2011). New CT response criteria in non-small cell lung cancer: proposal and application in EGFR tyrosine kinase inhibitor therapy. Lung Cancer.

[12] Mattonen SA, Palma DA, Haasbeek CJ (2013). Distinguishing radiation fibrosis from tumour recurrence after stereotactic ablative radiotherapy (SABR) for lung cancer: a quantitative analysis of CT density changes. Acta Oncol.

[13] Davnall F, Yip CS, Ljungqvist G (2012). Assessment of tumor heterogeneity: an emerging imaging tool for clinical practice?. Insights Imaging.

[14] Chicklore S, Goh V, Siddique M (2013). Quantifying tumour heterogeneity in ^18^F-FDG PET/CT imaging by texture analysis. Eur J Nucl Med Mol Imaging.

[15] Ganeshan B, Panayiotou E, Burnand K (2012). Tumour heterogeneity in non-small cell lung carcinoma assessed by CT texture analysis: a potential marker of survival. Eur Radiol.

[16] Lovinfosse P, Janvary ZL, Coucke P (2016). FDG PET/CT texture analysis for predicting the outcome of lung cancer treated by stereotactic body radiation therapy. Eur J Nucl Med Mol Imaging.

[17] Ravanelli M, Farina D, Morassi M (2013). Texture analysis of advanced non-small cell lung cancer (NSCLC) on contrast-enhanced computed tomography: prediction of the response to the first-line chemotherapy. Eur Radiol.

[18] Mattonen S, Palma DA, Haasbeek CJ (2013). Assessment of response after stereotactic ablative radiation therapy (SABR) for lung cancer: can advanced CT image feature analysis predict recurrence?. Int J Radiat Oncol Biol Phys.

[19] Garcia-Figueiras R, Goh VJ, Padhani AR (2013). CT perfusion in oncologic imaging: a useful tool?. AJR Am J Roentgenol.

[20] Zhao L, Guan W, Han Y (2014). Comparative study on CT perfusion parameters of different types of lung cancer before and after chemoradiotherapy. J Biol Regul Homeost Agents.

[21] Tacelli N, Santangelo T, Scherpereel A (2013). Perfusion CT allows prediction of therapy response in non-small cell lung cancer treated with conventional and anti-angiogenic chemotherapy. Eur Radiol.

[22] Strauch LS, Eriksen RO, Sandgaard M (2016). Assessing tumor response to treatment in patients with lung cancer using dynamic contrast-enhanced CT. Diagnostics (Basel).

[23] Ohno Y, Koyama H, Fujisawa Y (2016). Dynamic contrast-enhanced perfusion area detector CT for non-small cell lung cancer patients: Influence of mathematical models on early prediction capabilities for treatment response and recurrence after chemoradiotherapy. Eur J Radiol.

[24] van Elmpt W, Zegers CM, Das M (2014). Imaging techniques for tumour delineation and heterogeneity quantification of lung cancer: overview of current possibilities. J Thorac Dis.

[25] Baxa J, Vondrakova A, Matouskova T (2014). Dual-phase dual-energy CT in patients with lung cancer: assessment of the additional value of iodine quantification in lymph node therapy response. Eur Radiol.

[26] Baxa J, Matouskova T, Krakorova G (2016). Dual-Phase Dual-energy CT in patients treated with erlotinib for advanced non-small cell lung cancer: possible benefits of iodine quantification in response assessment. Eur Radiol.

[27] Li GJ, Gao J, Wang GL (2016). Correlation between vascular endothelial growth factor and quantitative dual-energy spectral CT in non-small-cell lung cancer. Clin Radiol.

[28] Schmid-Bindert G, Henzler T, Chu TQ (2012). Functional imaging of lung cancer using dual energy CT: how does iodine related attenuation correlate with standardized uptake value of 18FDG-PET-CT?. Eur Radiol.

[29] Chang YC, Yu CJ, Chen CM (2012). Dynamic contrast-enhanced MRI in advanced non small-cell lung cancer patients treated with first-line bevacizumab, gemcitabine, and cisplatin. J Magn Reson Imaging.

[30] Tao XL, Ouyang H, Wu N (2015). Preliminary study of semi-quantitative and quantitative dynamic contrast-enhanced MRI in evaluating the response to concurrent chem oradiotherapy in patients with non-small cell lung cancer. Zhonghua Zhong Liu Za Zhi.

[31] Nensa F, Stattaus J, Morgan B (2014). Dynamic contrast-enhanced MRI parameters as biomarkers for the effect of vatalanib in patients with non-small-cell lung cancer. Future Oncol.

[32] Zhang R, Wang ZY, Li YH (2016). Dynamic contrast-enhanced MRI to predict response to vinorelbine-cisplatin alone or with rh-endostatin in patients with non-small cell lung cancer and bone metastases: a randomised, double-blind, placebo-controlled trial. Lancet.

[33] de Langen AJ, van den Boogaart V, Lubberink M (2011). Monitoring response to antiangiogenic therapy in non-small cell lung cancer using imaging markers derived from PET and dynamic contrast-enhanced MRI. J Nucl Med.

[34] Khalil A, Majlath M, Gounant V (2016). Contribution of magnetic resonance imaging in lung cancer imaging. Diagn Interv Imaging.

[35] Koyama H, Ohno Y, Seki S (2013). Magnetic resonance imaging for lung cancer. J Thorac Imaging.

[36] Yabuuchi H, Hatakenaka M, Takayama K (2011). Non-small cell lung cancer: detection of early response to chemotherapy by using contrast-enhanced dynamic and diffusion-weighted MR imaging. Radiology.

[37] Xu HD, Zhang YQ, Shen WY (2017). Diffusion-weighted imaging in evaluating the efficacy of concurrent chemoradiotherapy in the treatment of non-small cell lung cancer. Tumori.

[38] Ohno Y, Koyama H, Yoshikawa T (2012). Diffusion-weighted MRI versus ^18^F-FDG PET/CT: performance as predictors of tumor treatment response and patient survival in patients with non-small cell lung cancer receiving chemoradiotherapy. AJR Am J Roentgenol.

[39] Wang LL, Lin J, Liu K (2014). Intravoxel incoherent motion diffusion-weighted MR imaging in differentiation of lung cancer from obstructive lung consolidation: comparison and correlation with pharmacokinetic analysis from dynamic contrast-enhanced MR imaging. Eur Radiol.

[40] Deng Y, Li X, Lei Y (2016). Use of diffusion-weighted magnetic resonance imaging to distinguish between lung cancer and focal inflammatory lesions: a comparison of intravoxel incoherent motion derived parameters and apparent diffusion coefficient. Acta Radiol.

[41] Albain KS, Swann RS, Rusch VW (2009). Radiotherapy plus chemotherapy with or without surgical resection for stage Ⅲ non-small-cell lung cancer: a phase Ⅲ randomised controlled trial. Lancet.

[42] Nakajima T, Shingyoji M, Anayama T (2016). Spectrum analysis of endobronchial ultrasound radiofrequency of lymph nodes in patients with lung cancer. Chest.

[43] He HY, Lv XD, Ma H (2016). Value of endobronchial ultrasound elastography in the diagnosis of mediastinal and hilar lymph node metastasis in lung cancer. Zhong Nan Da Xue Xue Bao Yi Xue Ban.

[44] Genestreti G, Burgio MA, Matteucci F (2015). Endobronchial/endoesophageal ultrasound (EBUS/EUS) guided fine needle aspiration (FNA) and ^18^F-FDG PET/CT scanning in restaging of locally advanced non-small cell lung cancer (NSCLC) treated with chemo-radiotherapy: a mono-institutional pilot experience. Technol Cancer Res Treat.

[45] Madsen PH, Holdgaard PC, Christensen JB (2016). Clinical utility of F-18 FDG PET-CT in the initial evaluation of lung cancer. Eur J Nucl Med Mol Imaging.

[46] Sheikhbahaei S, Mena E, Yanamadala A (2017). The value of FDG PET/CT in treatment response assessment, follow-up, and surveillance of lung cancer. AJR Am J Roentgenol.

[47] Yin N, Wang ZX, Zhu YB (2016). Clinical value of changes of SUV_max_ in series ^18^F-FDG PET/CT imaging before and after chemotherapy for non-small cell lung cancer. Zhonghua Yi Xue Za Zhi.

[48] Sawada S, Suehisa H, Ueno T (2016). Monitoring and management of lung cancer patients following curative-intent treatment: clinical utility of 2-deoxy-2-[fluorine-18]fluoro-d-glucose positron emission tomography/computed tomography. Lung Cancer (Auckl).

[49] Zukotynski KA, Gerbaudo VH (2017). Molecular imaging and precision medicine in lung cancer. PET Clin.

[b50] 50Wang JB. Nodal staging, natural growth and metabolic response to treatment evaluated with serial ^18^F-fluorodeoxyglucose positron emission tomography/computed tomography scans in radical radiotherapy candidates with non-small cell lung cancer: Peking Union Medical College Hospital; 2012.王静波. ^18^F-FDG PET/CT在非小细胞肺癌放射治疗中的应用及相关分析: 北京协和医学院; 2012.

[51] Ohri N, Duan F, Machtay M (2015). Pretreatment FDG-PET metrics in stage Ⅲ non-small cell lung cancer: ACRIN 6668/RTOG 0235. J Natl Cancer Inst.

[52] Usmanij EA, de Geus-Oei LF, Troost EG (2013). ^18^F-FDG PET early response evaluation of locally advanced non-small cell lung cancer treated with concomitant chemoradiotherapy. J Nucl Med.

[53] Satoh Y, Onishi H, Nambu A (2014). Volume-based parameters measured by using FDG PET/CT in patients with stage I NSCLC treated with stereotactic body radiation therapy: prognostic value. Radiology.

[54] Everitt SJ, Ball DL, Hicks RJ (2014). Differential (18)F-FDG and (18)F-FLT uptake on serial PET/CT imaging before and during definitive chemoradiation for non-small cell lung cancer. J Nucl Med.

[55] Szyszko TA, Yip C, Szlosarek P (2016). The role of new PET tracers for lung cancer. Lung Cancer.

